# Anti‐PD‐1/CTLA‐4 bispecific antibody‐based combination therapy for cervical cancer: a multicenter study with biomarker analysis

**DOI:** 10.3389/fimmu.2026.1967065

**Published:** 2026-10-01

**Authors:** Jiwei Li, Yuping Deng, Tao Wu, Chao Xu, Wen Zou, Jia Yao, Jingjing Wang

**Affiliations:** 1 Department of Oncology, The Second Xiangya Hospital, Central South University, Changsha, China; 2 Department of Gynecologic Oncology, The Affiliated Cancer Hospital of Xiangya School of Medicine, Hunan Cancer Hospital, Central South University, Changsha, China; 3 Department of Oncology, Changde Hospital, Xiangya School of Medicine, Changde, China; 4 Department of General Surgery, The Second Xiangya Hospital, Central South University, Changsha, China

**Keywords:** biomarkers, cervical cancer, immunotherapy, resistance, RNA sequence

## Abstract

**Background:**

Cadonilimab is a PD‐1/CTLA‐4 bispecific antibody with robust activity in platinum‐pretreated advanced cervical cancer, while its first‐line value and resistance mechanisms remain unclear.

**Methods:**

This multicenter retrospective study enrolled 52 treatment‐naive patients with recurrent/metastatic cervical cancer (R/M CC) receiving cadonilimab plus chemotherapy. We assessed clinical efficacy and safety, and performed RNA‐seq and immunohistochemistry for exploratory biomarker analysis.

**Results:**

The 2‐year overall survival, 2‐year progression‐free survival and objective response rate were 82.6%, 66.7% and 75.0%, respectively. Main treatment‐related adverse events included myelosuppression, thyroid dysfunction, gastrointestinal reactions and fatigue, demonstrating acceptable safety. Transcriptomic data showed suppressed Hippo, NF‐κB, RIG‐1 pathways and reduced M1‐macrophage polarization in patients with longer PFS. Candidate genes including GGT6 and FRG2 were identified as putative resistance‐associated markers in this small exploratory cohort. Higher M1‐macrophage polarization and p‐STAT1 expression were observed in short‐PFS patients, indicating a possible link between p‐STAT1 activation and M1‐macrophage enrichment. Of note, outcomes should be interpreted considering the retrospective single‐arm design and limited sample size.

**Conclusions:**

Cadonilimab‐containing regimen achieved promising efficacy and an acceptable safety profile as first‐line therapy for R/M CC. The biomarker findings are exploratory and provide preliminary evidence for future research on bispecific‐antibody resistance.

## Introduction

Cervical cancer (CC) is the fourth most common cancer among women worldwide (1, 2). The phase III KEYNOTE‐826 trial demonstrated that adding pembrolizumab to platinum‐based chemotherapy with or without bevacizumab significantly improved progression‐free and overall survival and reduced the risk of death (Hazard ratio, HR = 0.60) in patients with PD‐L1 CPS ≥ 1, recurrent, or metastatic cervical cancer (3, 4). These findings established pembrolizumab‐containing combination regimen as the first‐line standard‐of‐care for this patient population (3). However, the 2-year overall survival rate (OS) for recurrent or metastatic (R/M) CC patients with PD‐L1 CPS ≥ 1 is only 53.5% in KEYNOTE‐826 trial (4).

In addition to PD-1/L1 inhibitors, cytotoxic T lymphocyte associated antigen-4 (CTLA-4) inhibitors have also been widely studied and clinically applied as immune checkpoint inhibitors (ICI) (5). Combination therapy with PD‐1/L1 and CTLA‐4 inhibitors represents a promising therapeutic strategy supported by complementary immunological mechanisms and clinical evidence in selected tumor settings (6–9), but the incidence of severe adverse events is markedly increased (10). Consequently, the safety risks associated with dual immune checkpoint blockade could not be overlooked and represent a major bottleneck limiting its clinical application.

Cadonilimab (AK 104) is a pioneering bispecific monoclonal antibody targeting PD-1/CTLA-4. A phase II clinical trial investigated the efficacy and safety of cadonilimab in the later-line therapy for R/M CC (11). The ORR (Overall Response Rate) was 33.0% and the CRR (Complete Response Rate) was 12.0%, with a median OS of 17.5 months. Grade 3–4 treatment-related adverse events occurred in 28.8% of patients (11). Based on this study, Cadonilimab has been approved in China in 2022 for the later-line treatment of R/M CC patients with R/M CC (12). The phase 3 COMPASSION-16 trial evaluated the efficacy of first-line treatment with Cadonilimab or placebo in combination chemotherapy (cisplatin or carboplatin] plus paclitaxel), with or without bevacizumab in R/M CC (13, 14). The median OS was 28.8 months (95% CI, 26.3) in the cadonilimab group, compared to 25.3 months in the placebo group in patients who had metastatic disease at baseline (HR, 0.73; 95% CI, 0.52, 1.02) (13, 14). Bispecific monoclonal antibodies have shown promising prospects in the first-line treatment of R/M CC, but the efficacy and safety of cadonilimab for patients with cervical cancer in the real-world and the prognostic biomarker need further exploration.

Accordingly, this retrospective, multicenter, uncontrolled real-world study was performed to evaluate the feasibility, objective efficacy, survival outcomes, and safety profile of cadonilimab combined with chemotherapy ± bevacizumab as first-line treatment for R/M CC. Additionally, we conducted exploratory biomarker analysis via RNA-sequencing to identify potential prognostic and predictive factors associated with treatment response, and provide pragmatic real-world evidence to guide clinical decision-making and further biomarker-driven research for this patient population.

## Methods

### Patient

We retrospectively analyzed a cohort of patients diagnosed with metastatic/advanced cervical cancer between December 2022 and December 2023 at three hospitals: Second Xiangya Hospital of Central South University, People’s Hospital of Changde City, and Hunan Cancer Hospital. The inclusion criteria were as follows: (1) patients aged 18 years or older; (2) patients with recurrent or metastatic cervical cancer who had not received prior systemic therapy for metastasis (adjuvant or neoadjuvant therapy was permitted if > 12 months prior); (3) patients with complete follow-up data; and (4) patients who received combination therapy with ICIs and chemotherapy. The exclusion criteria were as follows: (1) tumors other than metastatic/advanced cervical cancer, infectious diseases, blood disorders, or severe hepatic or renal dysfunction; and (2) patients with diseases eligible for curative local treatment, previous PD-(L)1 inhibitor therapy, active central nervous system metastases, autoimmune diseases, immunodeficiency, or any other form of immunosuppressive therapy.

The final follow-up was conducted on 20 September 2025. All study procedures were performed in accordance with relevant guidelines and regulations and adhered to the Declaration of Helsinki. The study protocol was approved by the Ethics Committee of the Second Xiangya Hospital, Central South University, Changsha, China (Ethic number: LYEC2026-K0139).

### Treatment

All patients received treatment with bispecific antibody (cadonilimab) combined with chemotherapy ± bevacizumab. Chemotherapy protocols including paclitaxel (135–175 mg/m2) combined with carboplatin (AUC = 4–5)/cisplatin (50–70 mg/m2) and platinum monotherapy for up to 6 cycles every three weeks were selected according to patient renal function and physical status. Bevacizumab was administered at a dose of 10 mg/kg every three weeks until disease progression or intolerable toxicity occurred. The immunotherapy regimen consisted of cadonilimab (a bispecific antibody) administered at a dose of 10 mg/kg once every three weeks, continuing until disease progression or the onset of intolerable toxicity. Treatment suspension or permanent discontinuation may be implemented if tumor progression is observed, intolerable adverse events occur (e.g., severe myocarditis and pancreatitis), or a discontinuation decision is made by either the patient or attending physician. Palliative radiation therapy with intensity modulation is allowed, which is planned by using Varian Eclipse treatment planning system version 11.0 (Varian Medical Systems, Palo Alto, CA, USA) and performed with Varian 23EX (Varian Medical Systems) for 6-MV X-ray therapy.

### Data collection

Clinical data including age, performance status, metastatic sites, pathological differentiation, tumor PD-L1 status, treatment strategy, imaging findings (CT, MRI, or PET/CT), and adverse events were collected. Tumor responses were evaluated exclusively according to RECIST version 1.1. Given the retrospective nature of this multi‐center study, imaging examinations were not prospectively standardized for immune‐related response assessment, and consistent irRECIST evaluation could not be performed across all enrolled patients. During treatment, routine laboratory monitoring included complete blood counts, electrolytes, renal and liver function, myocardial enzymes, thyroid function, cortisol levels, and blood glucose. Post-chemotherapy follow-up was scheduled every 3 months for the first 2 years, every 6 months for years 3-5, and annually thereafter. At each visit, physical examination, imaging studies, and laboratory tests were performed as clinically indicated. Additionally, both overall adverse events (AEs) and immune‐related adverse events (irAEs) were documented according to the National Cancer Institute Common Terminology Criteria for Adverse Events, Version 5.0 (CTCAE v5.0) (15, 16). In this retrospective cohort, treatment‐related adverse events (TRAEs) were categorized according to event phenotypes and temporal onset. Hematologic toxicities and acute gastrointestinal reactions were predominantly attributed to chemotherapy. Immune‐related adverse events (irAEs) were identified based on typical ICI‐associated event patterns, clinical management (e.g., corticosteroid administration), and exclusion of alternative etiologies such as infection or chemotherapy‐induced injury. Notably, some adverse events (e.g., fatigue) are non‐specific and could not be definitively ascribed to cadonilimab‐mediated immune activation versus chemotherapy toxicity in this real‐world setting. Given the combination‐treatment design, many TRAEs may represent overlapping toxic effects from multiple therapeutic agents.

### Survival analysis

All statistical analyses were performed using GraphPad Prism 9.0 (GraphPad Software, San Diego, CA) and R version 4.3.1 (R Foundation for Statistical Computing). Primary endpoints included overall survival (OS), progression-free survival (PFS), and objective response rate (ORR). OS was defined as the interval from the start of first-line therapy to death from any cause or last follow-up. PFS was calculated from the start of first-line therapy to radiologically confirmed disease progression or censoring at last follow-up. Objective response rate (ORR) was assessed in accordance with RECIST v1.1 criteria, comprising complete response (CR) and partial response (PR), the latter defined as a ≥30% reduction in the sum of the longest diameters of target lesions sustained for ≥4 weeks.

Categorical variables were compared using Fisher’s exact test or Pearson’s χ² test, as appropriate. Survival probabilities were estimated via the Kaplan-Meier method, with between-group differences evaluated by log-rank tests. Univariate Cox proportional hazards regression models were first performed to preliminarily evaluate the association between each candidate variable and clinical survival endpoints. For multivariate Cox regression analysis, we adopted a clinically driven fully adjusted modeling strategy with the enter methods, where all clinically meaningful prognostic and therapeutic confounding factors were forced into the regression model simultaneously without preliminary filtering based on the P values derived from univariate analysis. The covariates incorporated into the multivariate model included established prognostic factors for recurrent/metastatic cervical cancer, such as patient age, pathological subtype, concomitant bevacizumab administration, radiotherapy. All categorical variables were coded as dummy variables for regression modeling. Hazard ratios (HRs) with corresponding 95% confidence intervals (CIs) and Wald P-values were calculated to quantify the independent predictive effect of each variable on PFS and OS. All statistical tests were two-sided, and P < 0.05 was defined as statistically significant.

### RNA‐sequencing

We screened a total of 52 patients with recurrent or metastatic cervical cancer who received first-line combination therapy incorporating cadonilimab. Thirty-three patients were excluded for the following reasons: unavailable formalin-fixed paraffin-embedded (FFPE) pretreatment tumor specimens (n=31), low tumor cellularity and compromised RNA integrity (n=2). The final RNA-seq cohort comprised 19 evaluable patients (**Supplementary Figure 1**). Transcriptome sequencing was performed following a standardized and previously validated experimental pipeline with minor modifications (17). Briefly, total RNA was isolated from formalin-fixed paraffin-embedded (FFPE) tumor tissue specimens using the MagPure FFPE RNA Extraction Kit (Cat. No. IVD4144, Magen Biotechnology, Guangzhou, China) according to the manufacturer’s column-based purification protocol. All experimental operations were conducted on ice to prevent RNA degradation, and residual genomic DNA contamination was eliminated via the built-in DNase I digestion step during RNA purification.

The concentration and purity of eluted total RNA were initially quantified using a Qubit 4.0 Fluorometer (Thermo Fisher Scientific, USA) with the Qubit RNA HS Assay Kit, and only RNA samples with a total amount ≥100 ng and A260/A280 ratio ranging from 1.8 to 2.2 were included for subsequent library construction. RNA integrity was further evaluated by capillary electrophoresis using the Qsep100 Bioanalyzer (BiOptic Inc., Taiwan, China). Samples with a DV200 value (the percentage of RNA fragments >200 bp) ≥40% were considered qualified and retained for downstream experiments, consistent with the standard quality control criteria for FFPE-derived RNA sequencing.

High-quality total RNA was subjected to ribosomal RNA (rRNA) depletion to eliminate the interference of abundant rRNA on transcriptome detection. The Hieff NGS^®^ MaxUp Human rRNA Depletion Kit (Cat. No. 12257ES24, Yeasen Biotechnology, Shanghai, China) was utilized to remove cytoplasmic and mitochondrial rRNA through specific probe hybridization and RNase H-mediated enzymatic digestion, followed by magnetic bead purification of target mRNA fragments.

Strand-specific dual-mode mRNA sequencing libraries were constructed using the Hieff NGS^®^ Ultima Dual-mode mRNA Library Prep Kit (Cat. No. 12309ES24, Yeasen Biotechnology, Shanghai, China) in strict accordance with the manufacturer’s instructions. Briefly, purified mRNA fragments were reverse-transcribed into first-strand and second-strand cDNA, followed by end repair, A-tail addition, adapter ligation, and PCR amplification to obtain qualified sequencing libraries. Library concentration and quality were verified via Qubit quantification and capillary electrophoresis prior to sequencing.

Qualified libraries were sequenced on the MGI DNBSEQ-T7 high-throughput sequencing platform (MGI Tech Co., Ltd., Shenzhen, China) with a paired-end 100 bp (PE100) sequencing strategy. Raw sequencing data were generated for subsequent transcriptome assembly, gene expression quantification, and differentially expressed gene screening.

### Functional exploration of differentially expressed genes

We categorized the 19 RNA‐seq patients into the responder group (CR + PR, n = 16) and non‐responder group (SD + PD, n = 3) according to treatment response to explore factors associated with tumor shrinkage.

Patients were stratified into short‐PFS (PFS < 6 months, early‐progressor/putative primary resistance, n = 2) and long‐PFS (PFS ≥ 6 months, sustained disease control, n = 17) groups according to a widely adopted 6‐month cutoff for defining early immunotherapy resistance from Society for Immunotherapy of Cancer (SITC) consensus recommendations for immune‐checkpoint inhibitor studies (18). This represents an exploratory empirical threshold instead of a statistically optimized cut‐off value. Standard multiple testing correction was used to control the false discovery rate (FDR) to avoid false positive results caused by excessive pairwise comparisons, which is a core step to ensure the reliability of DEG screening. Specifically, we used the widely accepted Benjamini-Hochberg (BH) method to calculate the adjusted FDR value for each gene, and to set strict screening criteria for DEGs: |log2 (fold change) | > 1 and FDR < 0.1. GO and KEGG enriched pathways were identified using the R package “clusterProfiler” with a *P* < 0.1 significance threshold (17). Computational deconvolution of RNA‐seq data was performed using the CIBERSORT algorithm with the LM22 signature matrix. Analyses were run with 1000 permutations, and quantile normalization was enabled.

### Prognostic analysis of the differentially expressed genes

We selected differentially expressed genes (DEGs) with the highest |log_2_FC| values and P < 0.05 from two pairwise comparisons: responder versus non‐responder groups, and short‐PFS versus long‐PFS groups, for subsequent survival analyses. For these selected genes, Kaplan‐Meier (KM) survival curves were generated using the R package survminer based on statistically‐derived optimal cut‐off values (19–21). Log‐rank tests were applied to evaluate survival differences between stratified groups.

### Single-cell RNA sequencing analysis

To dissect the cellular heterogeneity of key genes and tumor microenvironment in cervical cancer at the single-cell level, we performed single-cell RNA sequencing analysis on the dataset GSE224327 (22) using the “Seurat” package in R. We enrolled six patients with paired tumor tissues and adjacent non-tumor tissues for subsequent analyses. All six patients were diagnosed with cervical squamous cell carcinoma and received concurrent chemoradiotherapy, among whom three developed disease recurrence and three remained recurrence-free (22). A stringent quality control process was applied, retaining only cells that met the following criteria: expression of a minimum of 300 features, a mitochondrial gene percentage below 20%, a ribosomal gene expression percentage below 50%, and removal of contaminated cells using the decontX package. The filtered data were normalized using the LogNormalize method, and highly variable genes were selected for principal component analysis. Based on significant principal components, a shared nearest neighbor graph was constructed for cell clustering, with visualization achieved via Uniform Manifold Approximation and Projection (UMAP). Cell clusters were annotated by identifying specific marker genes and referencing established cell databases.

### Immunohistochemical staining

Hematoxylin and eosin staining and IHC studies were performed on formalin-fixed and paraffin-embedded tissue sections using standard methods. Primary antibodies against PD-L1 (clone number 22C3, Dako), CD68 (Abcam, ab213363), CD86 (Abcam, ab217266), STAT1 (Abcam, ab109320), and p-STAT1 (Abmart, TP56498S) (Ventana Medical Systems, USA) were applied on a BenchMark XT automated immunostainer (Ventana Medical Systems) with Cell Conditioning heat retrieval solution (Ventana Medical Systems).

### Evaluation of immunohistochemistry

The morphology and IHC results were reviewed by two pathologists (Dr. Zang HJ and Wang ZY), with the average value defined as the final score for each image. For every specimen, 10 random high-power fields (HPFs) were captured and examined. The proportion of immunopositive cells within each HPF was quantified and assigned a corresponding score. The immunohistochemical staining levels of CD68, CD86, STAT1 and phosphorylated STAT1 (p‐STAT1) were evaluated via the immunoreactivity score (IRS) system. The IRS value was calculated by multiplying the staining intensity score by the score for the percentage of positively stained cells. Staining intensity was graded from 1 to 4: 1, no staining; 2, weak staining; 3, moderate staining; 4, strong staining. The percentage of positively stained cells was also scored 1-4: 1, <5%; 2, 5%–25%; 3, 25%–50%; 4, ≥50%. Multiplying the two subscores yielded a total IRS ranging from 1 to 16. Samples with a total score <6 were classified as low expression (-), while those with a score ≥6 were categorized as high expression (+). PD-L1 expression was quantified using the combined positive score (CPS), which is defined as the count of PD-L1-stained tumor cells, lymphocytes, and macrophages divided by the total number of viable tumor cells, with the resulting quotient multiplied by 100.

## Result

### Patient characteristics

Detailed clinical characteristics, pathological features and treatment response of the enrolled patients are presented in **Table 1**. A total of 52 patients were included in the analysis, with a median body mass index (BMI) of 22.44 kg/m². The median follow-up time was 22 months (1–34 months). The median age of patients was 56.5 years (39 to 87 years). There were 29 cases of lung metastasis, 7 cases of liver metastasis, 11 cases of bone metastasis, 12 cases of pelvic metastasis, and 11 cases of lymph node metastasis. Regarding PD‐L1 expression, 39 patients (75%) had a CPS < 1, 4 patients (7.7%) had a CPS ≥ 1, and 9 patients (17.3%) had a CPS ≥ 10. Among the 52 enrolled patients, 23 received palliative radiotherapy and 34 were treated with bevacizumab. The median number of treatment cycles was 8 for cadonilimab and 5 for chemotherapy, respectively. No patient suspended or terminated treatment due to severe toxicities from chemotherapy or PD-1/CTLA4 bispecific antibody.

**Table 1:** Clinical characteristics and univariate analysis of overall survival, progression-free survival rates for patients received anti-PD-1/CTLA-4 bispecific antibody combined treatment.

Variable		Univariate analysis			
Clinicopathological parameters	Number (%)	2-year OS rate (%)	P-value	2-year PFS rate (%)	P-value
**Total**	52	82.6		66.7	
**Age, years**	Median:56.5				
<56.5	26	85.9		67.1	
≥56.5	26	80.1	0.701	66.0	0.983
**BMI**	Median:22.44				
<22.44	26	78.5		72.1	
≥22.44	26	87.2	0.536	61.3	0.624
Pathology					
Squamous carcinoma	42 (80.8)	86.5		66.0	
Adenocarcinoma	7 (13.5)	64.3	0.236	71.4	0.849
Adenosquamous carcinoma	3 (5.8)	66.7	0.267	66.7	0.867
Differentiation					
High	4 (7.7)	33.3	0.079	37.5	0.264
Medium	35 (67.3)	84.9		68.4	
Low	13 (25.0)	90.0	0.495	69.2	0.003
PD-L1 status					
CPS<1	39	82.2		66.7	
1≤CPS<10	4	75.0	0.634	75.0	0.820
CPS≥10	9	87.5	0.685	62.5	0.922
ECOG PS status					
0	3 (5.8)	100		50.0	
1	49 (94.2)	80.9	0.360	68.5	0.685
Chemotherapy cycles					
1-4	18 (34.6)	81.5		61.3	
5-8	34 (65.4)	83.6	0.643	69.5	0.538
Chemotherapy regimen					
Doublet agent	43 (82.7)	84.7		65.9	
Platinum alone	9 (17.3)	70.0	0.274	75.0	0.949
Palliative radiotherapy					
Yes	23 (44.2)	89.4		70.5	
No	29 (55.8)	74.0	0.261	61.4	0.505
Treated with Bevacizumab					
Yes	34 (65.4)	83.4		68.8	
No	18 (34.6)	80.1	0.815	62.1	0.721
Efficacy Evaluation					
CR	1	100		100	
PR	38	87.8		74.7	
SD	12	71.3		38.6	
PD	1	0.00	0.000	0.00	0.000

OS, Overall survival; PFS, Progression-free survival; BMI, Body Mass Index; ECOG PS, Eastern Cooperative Oncology Group performance status; CR, Complete response; PR, Partial response; PD, Progressive disease; SD, Stable disease.

### Prognostic analysis

The 2-year PFS and 2-year OS were 66.7% and 82.6%, respectively, and the ORR was 75.0% (**Figures 1A, B**; **Table 1**). As presented in **Figure 1**, the median progression-free survival (PFS) and overall survival (OS) were not reached. In the univariate prognostic analysis of all patients (**Table 1**), treatment response (p<0.001) was the only significant factor of OS in cervical cancer, while treatment response (p<0.001) and the degree of pathological differentiation (p=0.003) were found to be prognostic factors affecting PFS. Notably, PD-L1 expression showed no predictive value for overall survival (OS) or progression-free survival (PFS). Furthermore, the addition of bevacizumab (p>0.05) failed to confer survival benefits in patients with recurrent or metastatic (R/M) cervical cancer (**Table 1**).

**Figure 1:**
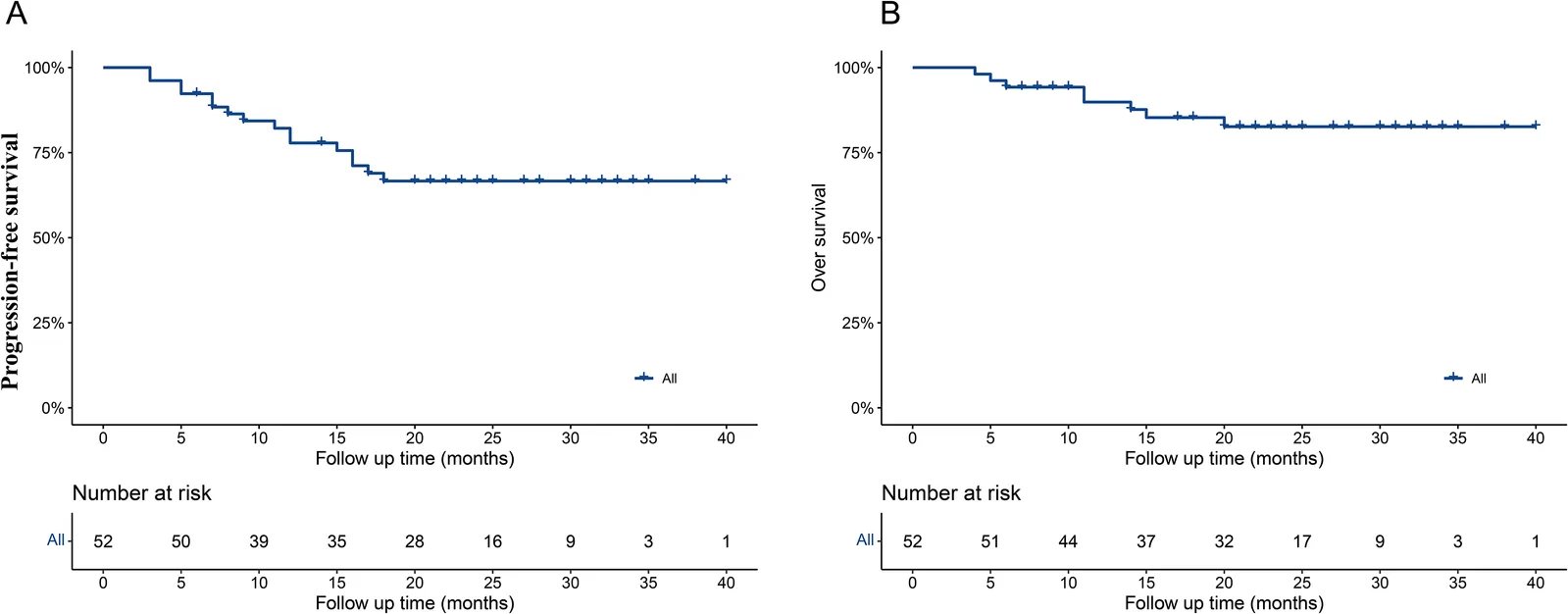
Survival analyses of patients with recurrent or metastatic cervical cancer (R/M CC) receiving first-line PD-1/CTLA-4 bispecific antibody (biAb) plus chemotherapy. **(A)** Kaplan-Meier curve for progression-free survival; **(B)** Kaplan-Meier curve for overall survival.

In terms of prognostic analysis, our study was limited to univariate analyses. Treatment response is a post-baseline endpoint, which is susceptible to immortal-time bias and was therefore not incorporated into multivariate Cox models. Owing to the small sample size and few statistically significant variables in univariate screening, multivariate prognostic modeling was not feasible.

### Toxicity

In total, 98.1% of the patients experienced adverse events, with no unexpected AEs recorded. The most common treatment-related adverse events (TRAEs) were neutropenia (82.7%), anemia (86.5%), nausea and vomiting (80.8%), fatigue (71.2%), thrombocytopenia (51.9%) and hypothyroidism (50%) (**Table 2**; **Supplementary Tables 2**, **3**). The incidence of all‐grade 3‐4 TRAEs was 32.7%, and the incidence of grade 3‐4 hematologic toxicities was 28.8%. IrAEs (Immune-Related Adverse Events) occurred in 35 (67.3%) patients (**Supplementary Table 3**), with the most frequent being hypothyroidism (26 [50.0%]), followed by rash (8 [15.4%]), infusion reaction (4 [7.7%]) and hepatotoxicity (4 [7.7%]). Two (3.8%) patients developed grade 3 and were successfully managed with therapeutic interventions.

**Table 2:** Adverse event profiles of cadonilimab‐based combination therapy for R/M cervical cancer.

Adverse effect	Bispecific antibody group (n=52)
**Hematologic**	51 (98.1)
G3-4	15 (28.8)
Neutropenia	43 (82.7)
G3-4	13 (25.0)
Thrombocytopenia	27 (51.9)
G3-4	7 (13.5)
Anemia	45 (86.5)
G3-4	3 (5.8)
Nonhematologic	
Nausea/vomiting	42 (80.8)
Hepatotoxicity	17 (32.7)
Rash	8 (15.4)
Infusion reaction	5 (9.6)
Hypothyroidism	26 (50.0)
G3-4	2 (3.8)
Fatigue	37 (71.2)

R/M, recurrent/metastatic; G, Grade.

### RNA sequencing results

The raw data available in the Zenodo database (https://zenodo.org/records/19287491, DOI: 10.5281/zenodo.19287491). We collected FFEP samples from 19 patients who received only cadonilimab plus chemotherapy and performed RNA-Seq analysis. To explore the key genes and biological pathways related to disease prognosis, we categorized the 19 patients into the short PFS (<6 months) group and long PFS (≥6 months) group. Compared with short PFS group, a total of 56 DEGs were identified in the long PFS group, including 21 upregulated and 35 downregulated genes (**Figures 2A, B**).

**Figure 2:**
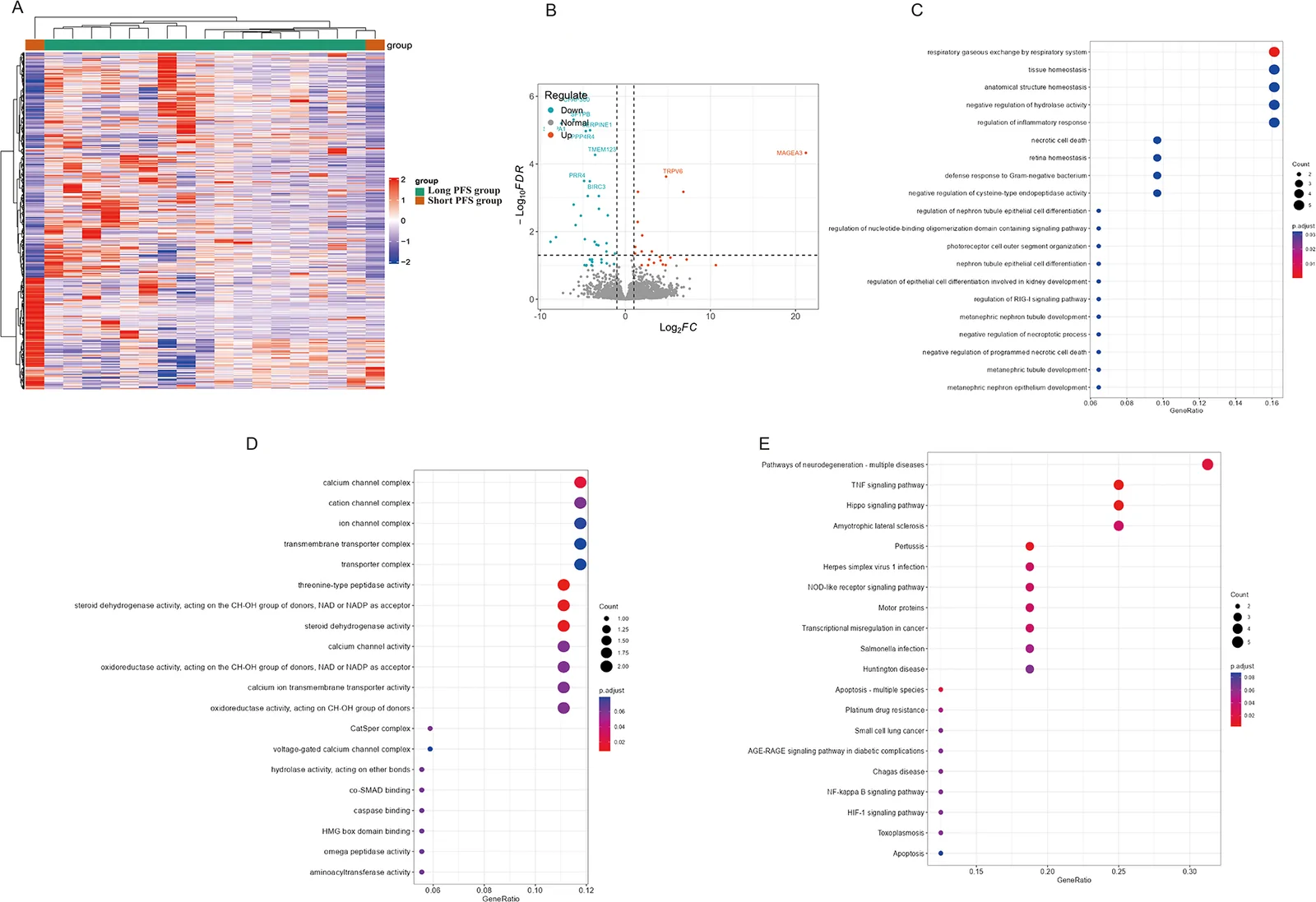
Transcriptomic differential expression profiling and functional enrichment analysis of patients stratified by short versus long progression-free survival (PFS). **(A)** Hierarchical clustering heatmap illustrating the expression patterns of differentially expressed genes (DEGs) between the short PFS cohort and long PFS cohort. Red represents upregulated genes, and blue represents downregulated genes. **(B)** Volcano plot displaying DEGs filtered under the threshold false discovery rate (FDR) < 0.1 between the two PFS subgroups. **(C)** Bubble plot of significantly suppressed GO signaling pathways enriched in the long PFS cohort compared to short PFS cohort. **(D)** Bubble plot of significantly activated GO signaling pathways enriched in the long PFS cohort compared to short PFS cohort. **(E)** Bubble plot of markedly downregulated KEGG signaling pathways enriched in the long PFS cohort compared to short-PFS cohort.

We characterized core biological pathways and functional modules potentially associated with treatment response and prognosis in this exploratory RNA‐seq subcohort. GO enrichment showed downregulation of the RIG-1 signaling pathway in patients with extended PFS (**Figure 2C**). By contrast, upregulated GO terms in this favorable prognosis group encompassed ion channels, transmembrane transporter complexes, and steroid dehydrogenase-related functions (**Figure 2D**). KEGG enrichment analysis identified several major suppressed signaling pathways in the long-PFS cohort versus the short-PFS cohort, including Hippo, NF-κB, TNF and HIF-1 signaling (**Figure 2E**).

Among these patients, 16 of them were in the responder (CR+PR) group, while 3 were in the non-responder (SD+PD) group. The profiling of the differentially expressed genes (DEGs) between the two groups was exhibited in a clustered heat map (**Supplementary Figure 2**). Compared with the non-responder group, only 3 DEGs were identified in the responder group, including two upregulated and one downregulated gene (**Supplementary Figure 2**). Given the scarcity of differentially expressed genes identified between the two cohorts, we did not conduct subsequent Gene Ontology (GO) and Kyoto Encyclopedia of Genes and Genomes (KEGG) enrichment analysis.

### Prognostic analysis of key genes across the entire cohort and database

We investigated the prognostic associations of the top 60 DEGs (30 up‐regulated and 30 down‐regulated genes) identified across different subgroups (**Supplementary Figure 3**; **Supplementary Table 1**). As shown in **Supplementary Table 1**, GGT6 and PRR4 had significant differences in PFS, while ALOX12 and MYT1L exhibited a noticeable trend. In the subsequent OS analysis, C1orfB7 and FRG2 showed statistical significance, while H1_7, MYT1L and DPYBL5 exhibited a noticeable trend (**Supplementary Table 1**).

We leveraged the TCGA database to provide external biological context for the prognostic relevance of our identified candidate biomarkers, rather than for direct validation under cadonilimab‐based therapy. The results showed that high ALOX12 expression was associated with favorable survival outcomes in cervical cancer, whereas high PRR4 expression correlated with poorer survival (**Figure 3**).

**Figure 3:**
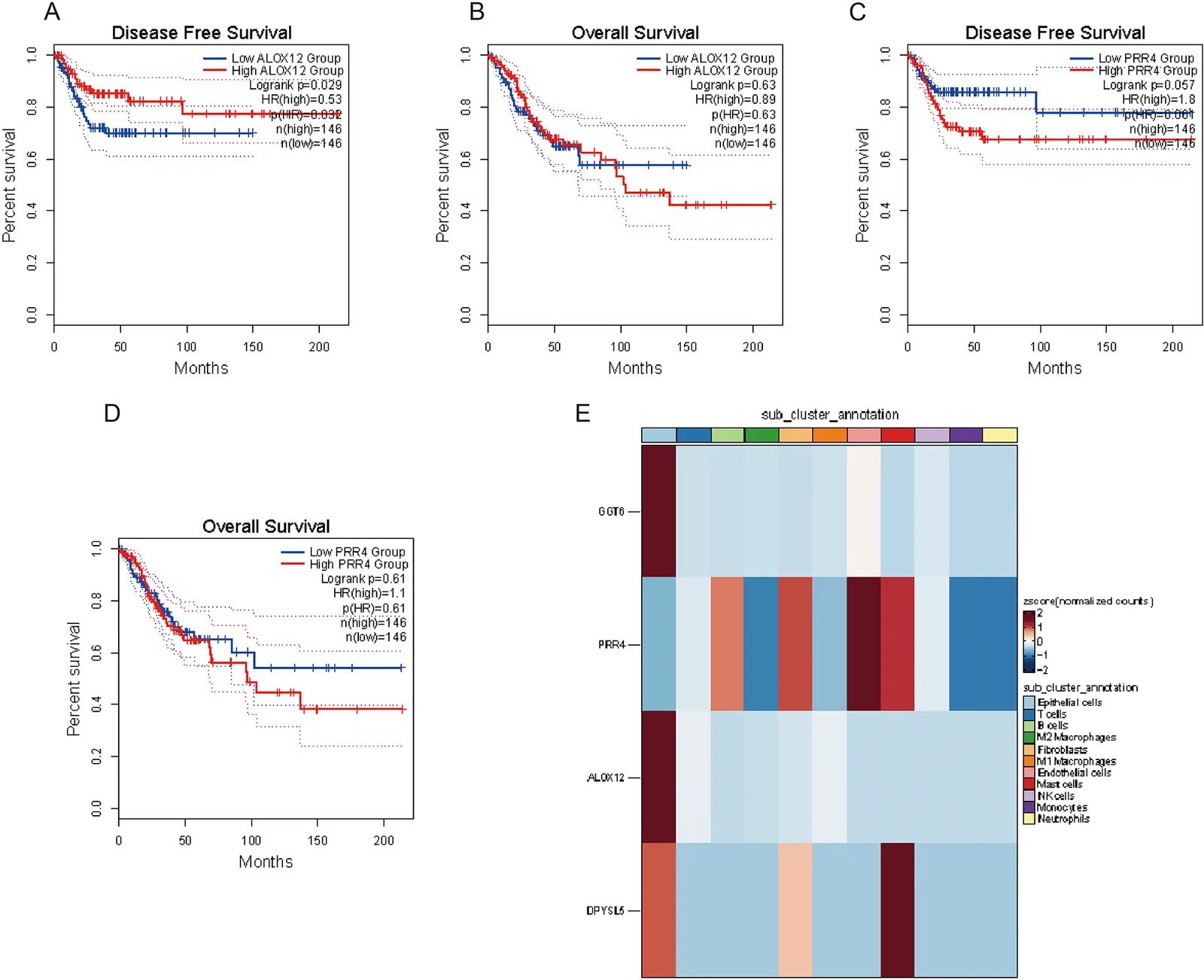
Prognostic validation of survival and single-cell expression localization of key genes in cervical cancer. **(A, B)** Kaplan -Meier analyses of disease-free survival (DFS, **(A)**) and overall survival (OS, **(B)**) for ALOX12 in cervical cancer patients based on the TCGA database. **(C, D)** Kaplan -Meier analyses of DFS **(C)** and OS **(D)** for PRR4 in cervical cancer patients based on the TCGA database. **(E)** Single-cell RNA sequencing analysis reveals the specific cellular origin and expression distribution of dysregulated genes in cervical cancer tissues.

To characterize the cellular origins of the most markedly dysregulated genes, we further interrogated public single‐cell RNA‐sequencing (scRNA‐seq, GSE224327) datasets to infer the cell populations driving their expression. As illustrated in **Figure 3E**, in cervical cancer tissues, GGT6 is predominantly expressed by epithelial cells; PRR4 is mainly detected in endothelial cells, mast cells, and fibroblasts; ALOX12 is largely localized to epithelial cells; and DPYBL5 is expressed in both epithelial cells and mast cells.

### The association of immune checkpoints and tumor microenvironment with treatment response

Given the established roles of immune checkpoint molecules and tumor microenvironment (TME) composition in modulating immunotherapy response, we systematically evaluated the expression of PD-L1, CTLA-4, TIM-3, IDO1, IDO2, LAG-3, and PD-1 across clinical subgroups. Transcriptomic analysis revealed no statistically significant differences in checkpoint expression between the short PFS and the long PFS cohorts (**Supplementary Figure 4**), nor between the responder group and the non-responder group (all *p* > 0.15, Wilcoxon test) (**Supplementary Figure 5**).

To characterize TME heterogeneity, we performed computational deconvolution analysis of RNA-seq data using the CIBERSORTx algorithm. Although the overall immune cellular landscape exhibited limited intergroup heterogeneity (Bray–Curtis similarity index > 0.85), neutrophil abundance was significantly elevated in non-responders relative to responders. Meanwhile, patients with short progression-free survival (PFS) displayed prominent enrichment of M1-type polarized macrophages versus those with long PFS (Mann–Whitney U test, p < 0.05) (**Figure 4**). Single-cell sequencing analysis (GSE224327) revealed that the expression profiles of macrophages, epithelial cells, and T cells within the tumor immune microenvironment of cervical cancer were significantly correlated with tumor recurrence (**Supplementary Figure 6**).

**Figure 4:**
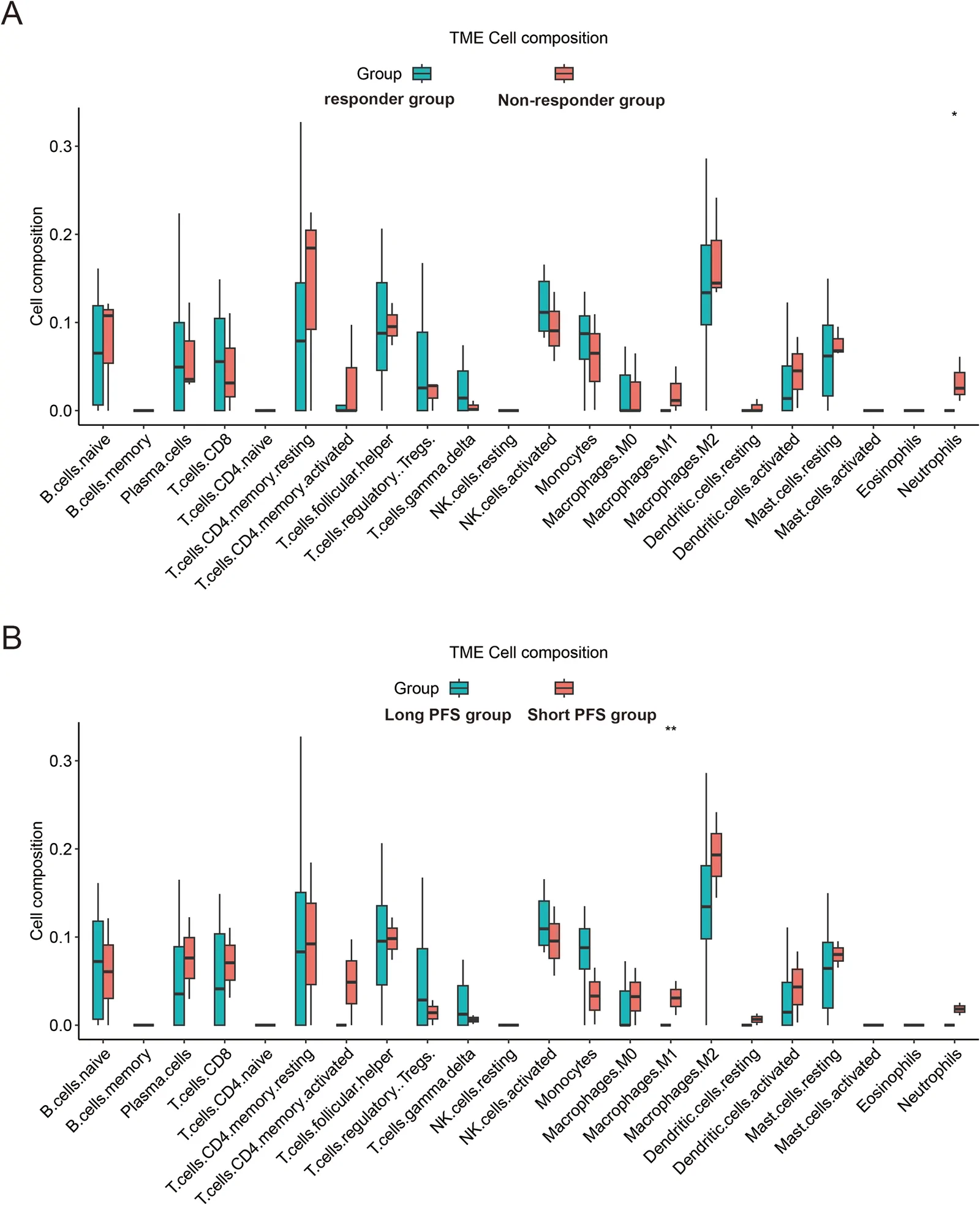
Landscape of tumor immune cell infiltration in different clinical subgroups of R/M CC patients receiving first-line PD-1/CTLA-4 bispecific antibody combination therapy. **(A)** Comparison of overall immune cell composition between responders and non-responders. **(B)** Comparison of overall immune cell composition between short-PFS and long-PFS patient subgroups.

### Immunohistochemical results

As described above, M1-type macrophages, RIG-1 signaling pathway, TNF signaling pathway, NF-κB signaling pathway and HIF-1α signaling pathway in cervical cancer tissues may exhibit significant correlation with the therapeutic response to PD-1/CTLA4 bispecific antibodies. To explore transcriptomic alterations at the protein level and investigate potential mechanisms underlying M1 macrophage polarization, we examined the pan‐macrophage marker CD68, the M1‐specific marker CD86, as well as total STAT1 and phosphorylated STAT1 (p‐STAT1) in this exploratory study.

As shown in **Figure 5**, CD68 is localized within the cytoplasm of tumor-associated macrophages and exhibits granular, brown staining in cervical cancer specimens. CD86 immunoreactivity is characterized by a granular brownish pattern and membrane staining in cervical cancer specimens. STAT1 immunoreactivity is characterized by granular brown staining distributed in the cytoplasm and nucleus of macrophages in cervical cancer specimens. Phosphorylated STAT1 (p-STAT1) immunoreactivity is characterized by intense granular brown nuclear staining with faint cytoplasmic signals in cervical cancer specimens (**Figures 5A–H**). Our results demonstrated that p‐STAT1 expression was significantly elevated in patients with short PFS compared to those with long PFS. Furthermore, IHC analysis revealed that CD86 expression was markedly lower in the long‐PFS cohort relative to the short‐PFS cohort (**Figures 5I, J**). The IHC staining intensities were strongly correlated with transcriptomic profiles from RNA‐seq, verifying the reliability and accuracy of our RNA‐seq data.

**Figure 5:**
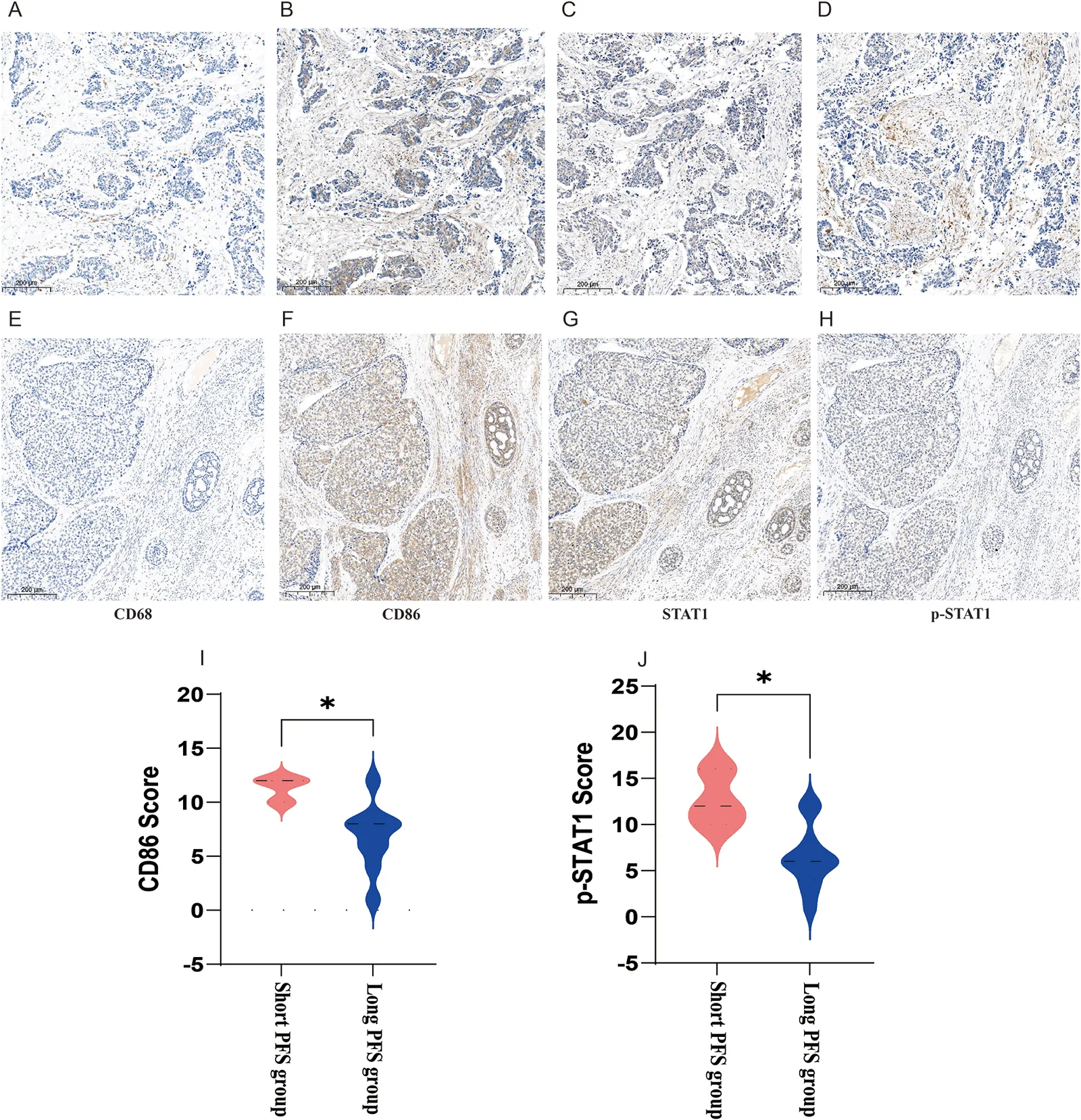
Immunohistochemical validation of STAT1 and M1 macrophage markers in clinical tumor tissues. **(A–D)** Representative immunohistochemical staining images of CD68, CD86, STAT1, and phosphorylated STAT1 (p-STAT1) derived from a single patient specimen (original magnification: ×100). **(E–H)** Immunohistochemical staining for CD68, CD86, STAT1, and p-STAT1 in tumor tissue from an independent patient (original magnification: ×100). **(I)** Correlation between CD86 expression levels and progression-free survival (PFS). **(J)** Association of p-STAT1 expression with patient PFS. *represents p<0.05.

## Discussion

In this real‐world retrospective cohort, cadonilimab combined with chemotherapy ± bevacizumab demonstrated encouraging clinical activity and an acceptable safety profile as first‐line therapy for recurrent or metastatic cervical cancer. In addition, we performed exploratory RNA‐sequencing analysis to identify candidate prognostic biomarkers linked to treatment response. Owing to the limited sample size of the RNA‐seq subcohort, these observations remain preliminary and can only provide a foundation for future research investigating bispecific antibody resistance in cervical cancer.

The clinical efficacy of cadonilimab administered as first-line or second-line treatment for recurrent and refractory cervical cancer has been substantiated by a series of landmark clinical trials and real-world investigations (**Table 3**) (14, 23–27). A phase II trial evaluated the efficacy and safety of QL1706, bifunctional Mabpair product of anti-PD-1 IgG4 and anti-CTLA-4 IgG1 antibodies, combined with chemotherapy ± bevacizumab as first-line treatment for R/M CC. The results showed that the ORR and mPFS were 75.9% and 14.3 months (28). Another phase II trial evaluated the efficacy and safety of bispecific antibodies (cadonilimab) combined with chemotherapy ± bevacizumab as first-line treatment for R/M CC and the ORR was 66.7-92.3% (25). A phase III clinical trial of cadonilimab for first-line treatment of R/M CC is also currently underway. Herein, we conducted this multicenter retrospective real-world study to assess the clinical efficacy and safety of cadonilimab-containing combination regimens for first-line R/M CC. Our cohort achieved encouraging 2-year OS, 2-year PFS, and ORR of 82.6%, 66.7%, and 75.0%, respectively, which were generally consistent with previously reported clinical results (25). Notably, consistent with the heterogeneous treatment designs of prior studies, all patients in our study received chemotherapy as backbone treatment, and partial cases were further supplemented with bevacizumab or radiotherapy. Given the retrospective study, limited sample size, and variable treatment modalities, the promising survival outcomes observed in our work should be interpreted cautiously and are considered the comprehensive benefit of multi-modal combination therapy.

**Table 3:** Summarization of cadonilimab-combined therapy for R/M cervical cancer.

Study	Number	ORR	CR	mPFS	mOS
COMPASSION-16 (13)	222	82.9%	35.6%	12.7m	Not reached
Chen et al. (23)	129	38.8%	8.0%	12.4m	Not reached
Pan et al. (26)	101	59.41%	4.0%	7.97m	8.90m
Ge et al. (24)	25	76.0%	24.0%	Not reached	Not reached
Wang et al. (27)	50	54.0%	12.0%	20.2m	Not reached
COMPASSION-13 (25)	45	73.3%	11.1%	N/A	Not reached

In terms of toxicity, the most common treatment-related adverse events (TRAEs) were neutropenia, anemia, thrombocytopenia, thyroid dysfunction, nausea and vomiting, and fatigue in this study. Grade 3 or 4 adverse events (AE) were reported in 32.7% of patients in this study, mainly hematological toxicity. A phase II trial of cadonilimab showed that the rate of treatment-related serious AEs was 51.1%, and grade ≥3 TRAEs were reported in 33 (73.3%) patients (25). The common adverse events included anemia, neutropenia, decreased appetite, thrombocytopenia, hypoesthesia, rash, etc. The occurrence of adverse events is relatively consistent. The adverse events of the combination of monoclonal and dual targeted immunotherapy in the first-line treatment were similar, including hematologic toxicity, neurotoxicity, gastrointestinal toxicity, and immune related adverse events. Cadonilimab demonstrated controllable safety for first-line treatment in cervical cancer.

Multiple genes have been proven to be associated with immunotherapy resistance and survival outcome in cervical cancer (29–31). A recent study identified a prognostic model for immunotherapy response using m6A/m5C/m1A-related genes (IQGAP3, PTBP1, STAC3, CUX1, SLC2A1, and CA2, etc.) (29). The prognostic model for bispecific antibody has not been investigated yet. In our study, our results indicated that some important genes such as GGT6, FRG2, and PRR4 may represent hypothesis‐generating candidate markers associated with therapeutic resistance; however, these findings remain preliminary and require further validation in larger independent patient cohorts.

The tumor microenvironment (TME) plays a critical role in shaping the response to immunotherapy. Key TME components, including cancer-associated fibroblasts (CAFs), Neutrophils, tumor-associated macrophages (TAMs), and myeloid-derived suppressor cells (MDSCs), modulate the microenvironment in ways that contribute to the development of immunotherapy resistance (32–36). Modulating TAM polarization and inhibiting tumor-associated neutrophils could sensitize cancer cells to immunotherapy (37). Our results demonstrate that high levels of neutrophils and macrophages correlate with treatment response and survival outcomes in this cohort. Considering the small RNA‐seq subcohort, these findings are exploratory. Hence, targeting these cells may hypothetically help overcome resistance to cadonilimab in cervical cancer, and further validation is warranted.

Previous studies have demonstrated that NF-κB was the central transcriptional effector of M1 polarization (38). TNF and HIF-1 signaling act as upstream modulators to activate or amplify NF-κB signaling (39, 40). STAT1 phosphorylation is the prerequisite for M1 macrophage polarization (41). Our study revealed activation of STAT1 phosphorylation in the short‐PFS subgroup of this cohort. As a shared downstream effector of the above differential pathways, p‐STAT1 may bridge transcriptomic pathway changes, macrophage polarization, and clinical outcomes. While these immunohistochemical results offer in‐situ histological support for our RNA‐seq and immune‐infiltration findings, they should be regarded as exploratory given the limited RNA‐seq subcohort size and need further validation in larger cohorts.

This study had several limitations. Firstly, this is a retrospective study. Secondly, the number of patients included is relatively small. Thirdly, there is a lack of long-term follow-up results. In addition, as this is a retrospective multi‐center study, it was difficult to reliably identify potential pseudoprogression events based on retrospective imaging data. Larger‐scale studies with longer follow‐up, as well as randomized clinical trials, are needed to confirm the present results.

In summary, this study indicated that the combination of cadonilimab and chemotherapy ± bevacizumab as first-line treatment for R/M CC is feasible and safe. RNA-seq analysis indicated interferon (IFN) pathway downregulation in treatment responders and patients with long PFS, and several key genes including GGT6 and FRG2 that were potentially associated with cadonilimab resistance were also screened out. Of note, the gene expression analysis in this study was restricted to a small patient subset, with a limited number of non-responder patients enrolled. Accordingly, these molecular findings should be regarded as preliminary exploratory hypotheses rather than definitive conclusions, and further validation in a larger and independent patient cohort is required before drawing firm conclusions regarding the relevant resistance mechanisms. Collectively, first-line cadonilimab combined with chemotherapy achieves encouraging clinical efficacy and exhibits an acceptable safety profile in our cohort. However, the retrospective uncontrolled study design, small sample cohort, and heterogeneous background therapies restrict our capacity to robustly verify the independent therapeutic effect of cadonilimab. Even so, these exploratory preliminary data on potential resistance mechanisms uncover novel therapeutic targets and provide a basis for further systematic research on bispecific antibody resistance in cervical cancer.

## Data Availability

The datasets presented in this study can be found in online repositories. The names of the repository/repositories and accession number(s) can be found in the article/**Supplementary Material**.
