# Supplementary material for: Anti‐PD‐1/CTLA‐4 bispecific antibody‐based combination therapy for cervical cancer: a multicenter study with biomarker analysis
Source: Front Immunol. 2026 Oct 1;17:1967065. doi: 10.3389/fimmu.2026.1967065 (PMC13669150; doi:10.3389/fimmu.2026.1967065)
Supplement: Supplementary file 1 [file DataSheet1.pdf]

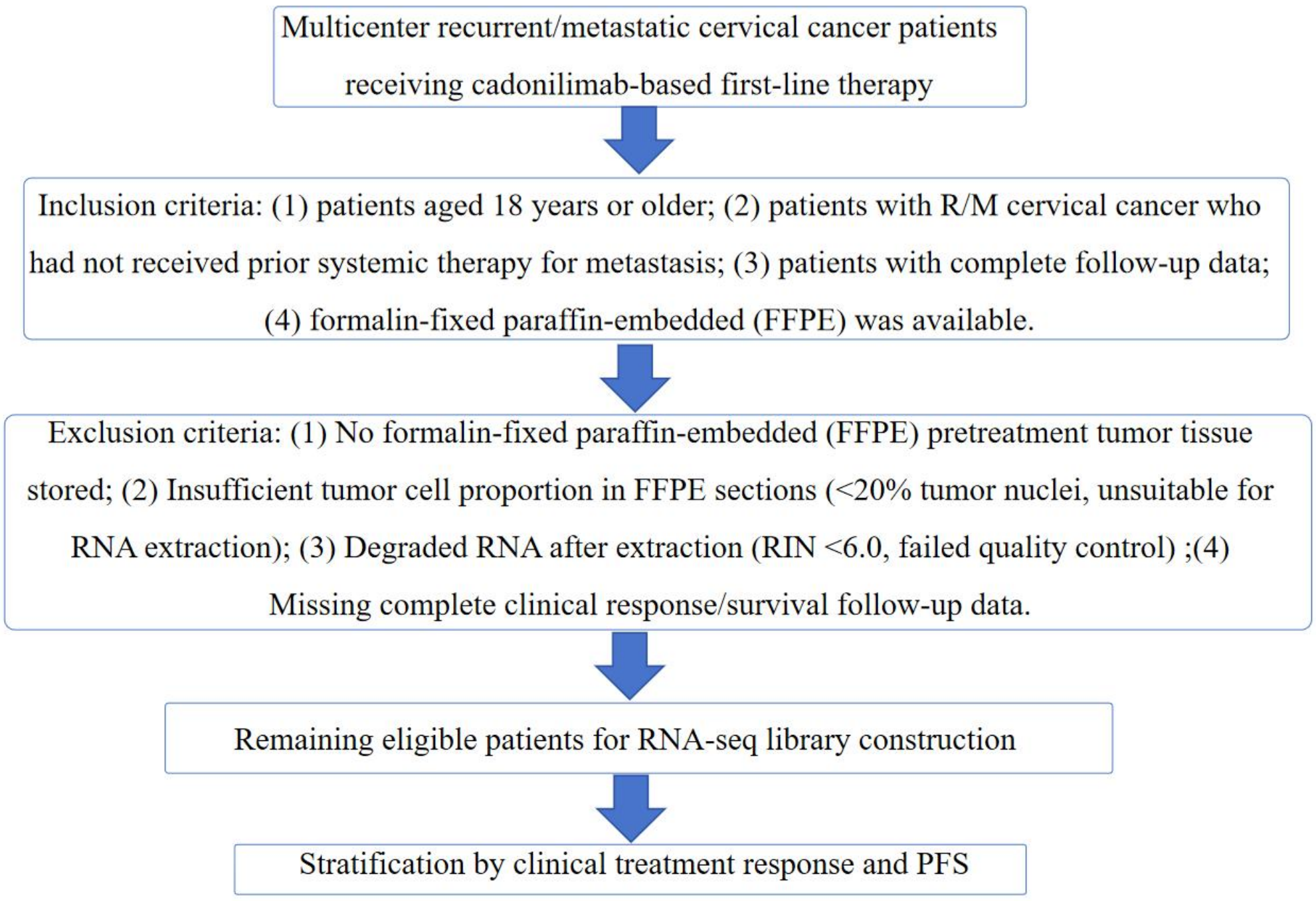

Supplementary figure 1. CONSORT-style patient flow diagram illustrating the screening, exclusion, and final enrollment process of the RNA-seq subcohort. A total of 52 patients with recurrent/metastatic cervical cancer receiving first-line cadonilimab-containing combination therapy were screened. Thirty three patients were excluded due to lack of preserved pretreatment FFPE tumor tissues (n=31), low tumor cellularity, poor RNA integrity (n=2), or incomplete clinical follow-up data.

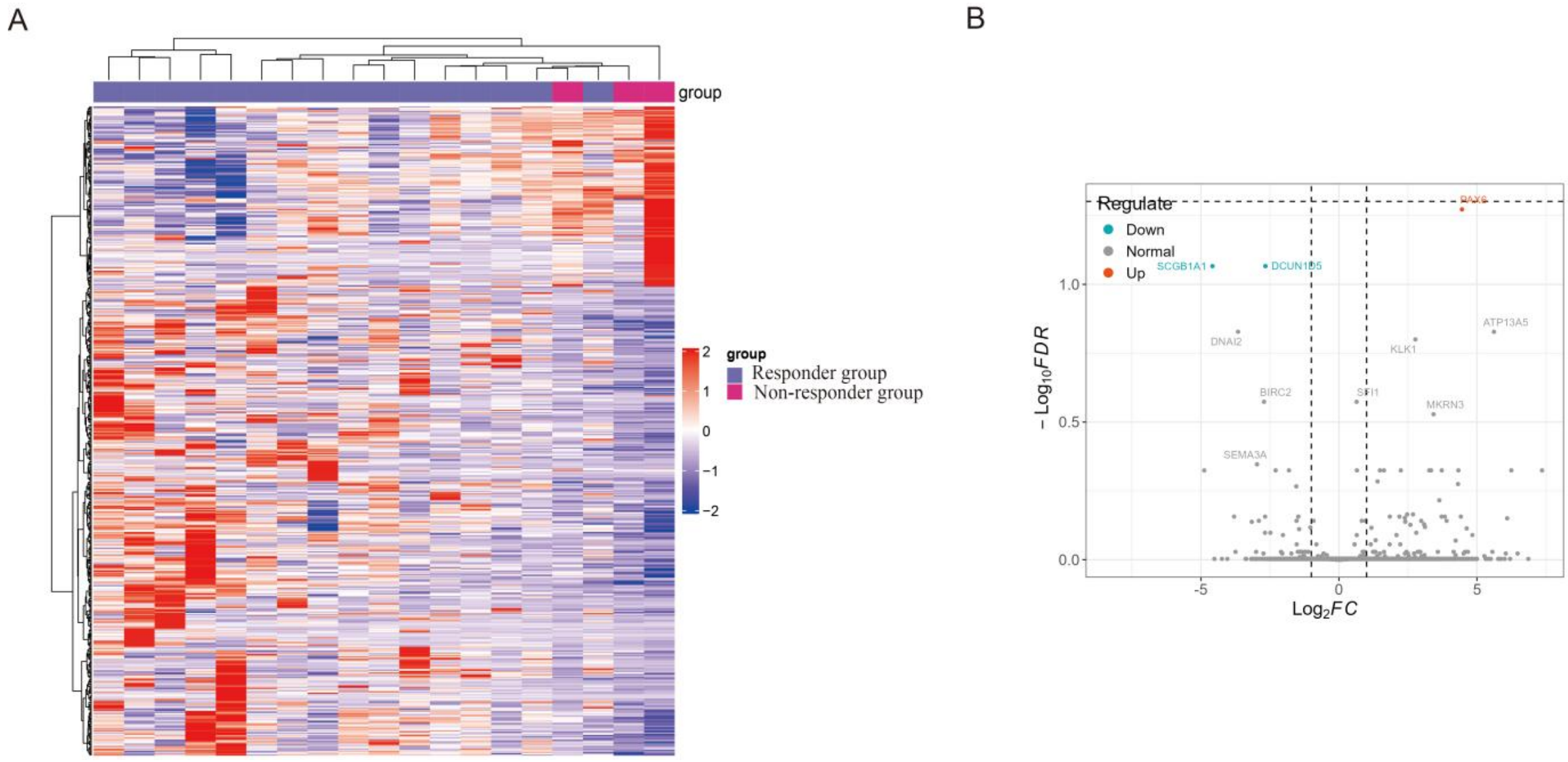

Supplementary Figure 2. Transcriptomic differential expression profiling of patients stratified by treatment response. (A) Hierarchical clustering heatmap illustrating the expression patterns of differentially expressed genes (DEGs) between the responder (CR+PR) group and the non-responder (SD+PD) group. Red represents upregulated genes, and blue represents downregulated genes. (B) Volcano plot displaying DEGs filtered under the threshold false discovery rate (FDR) < 0.1 between the two subgroups.

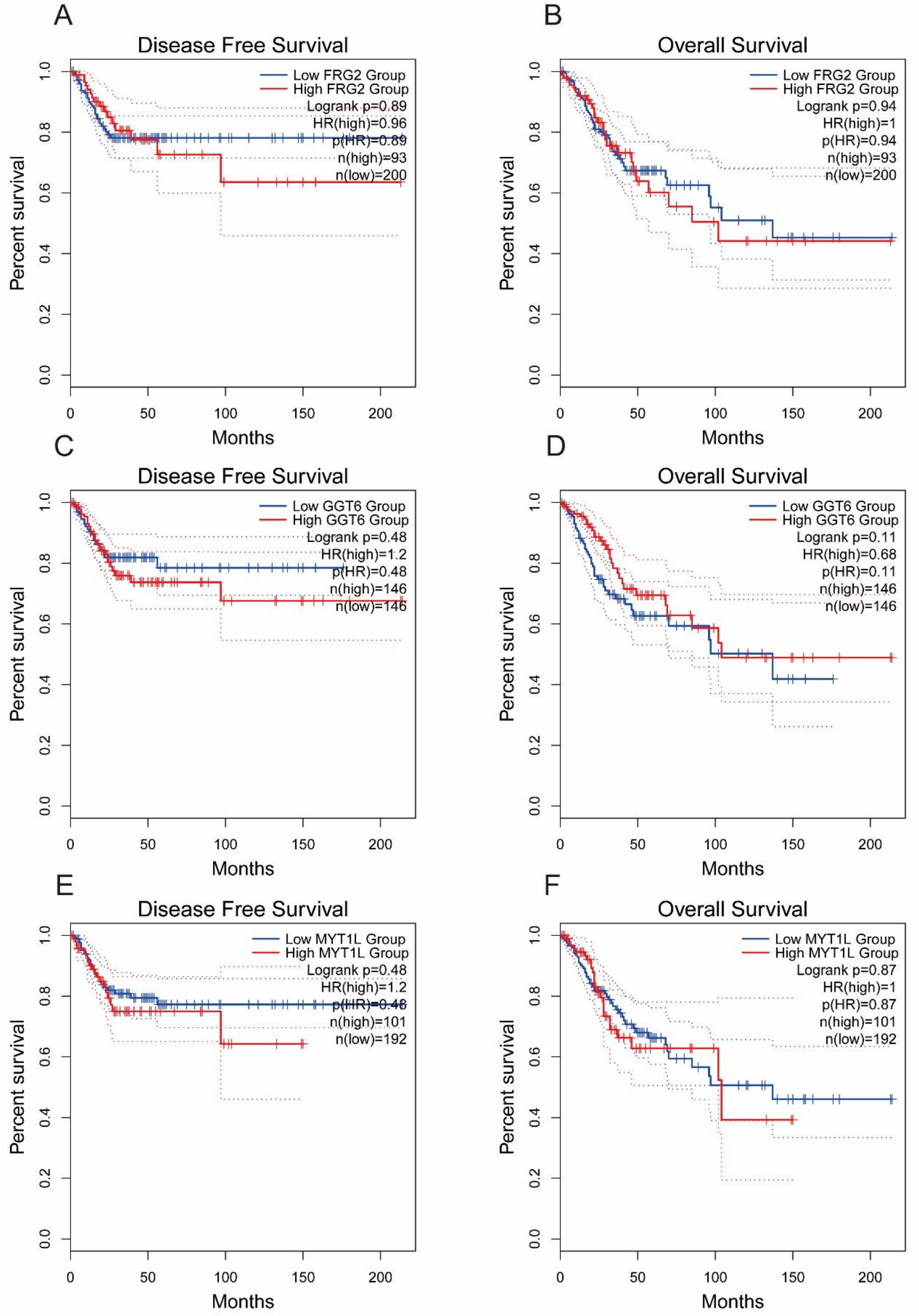

Supplementary figure 3. Prognostic analysis of FRG2, GGT6 and MYT1L in cervical cancer in the TCGA database.

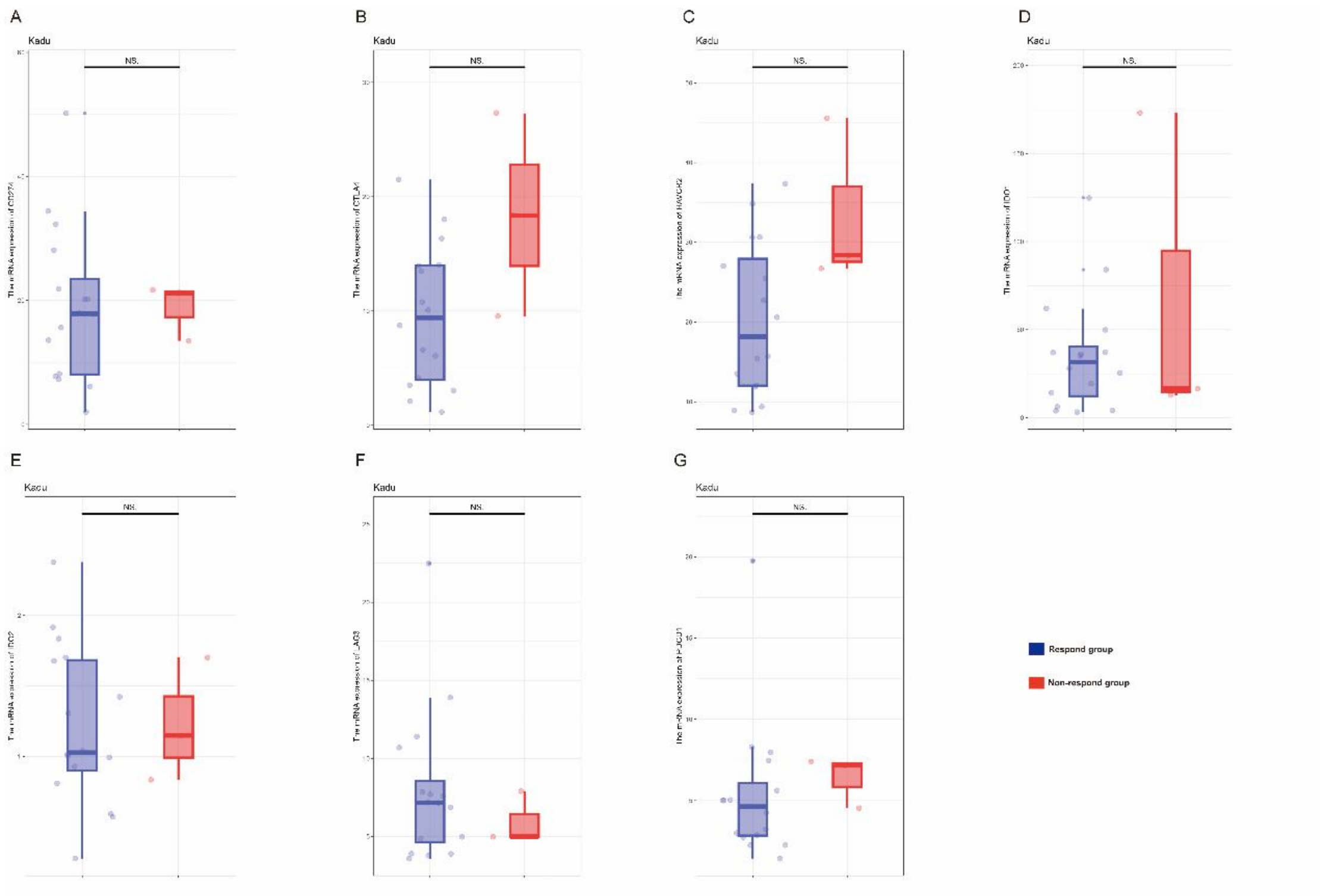

Supplementary figure 4. The expression of immune checkpoints between the short PFS group and the long PFS group.

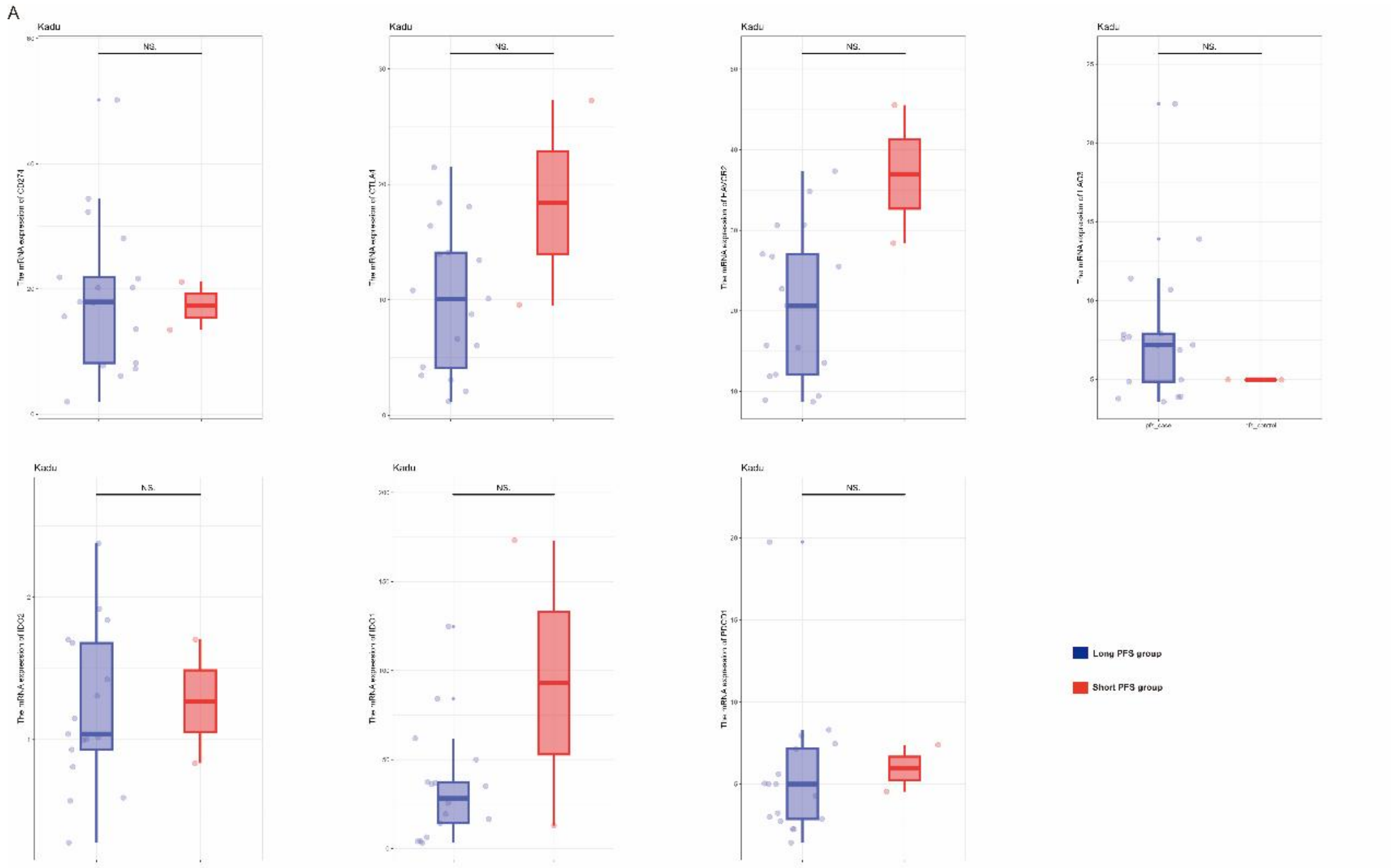

Supplementary figure 5. The expression of immune checkpoints between the responder group and the non-responder group.

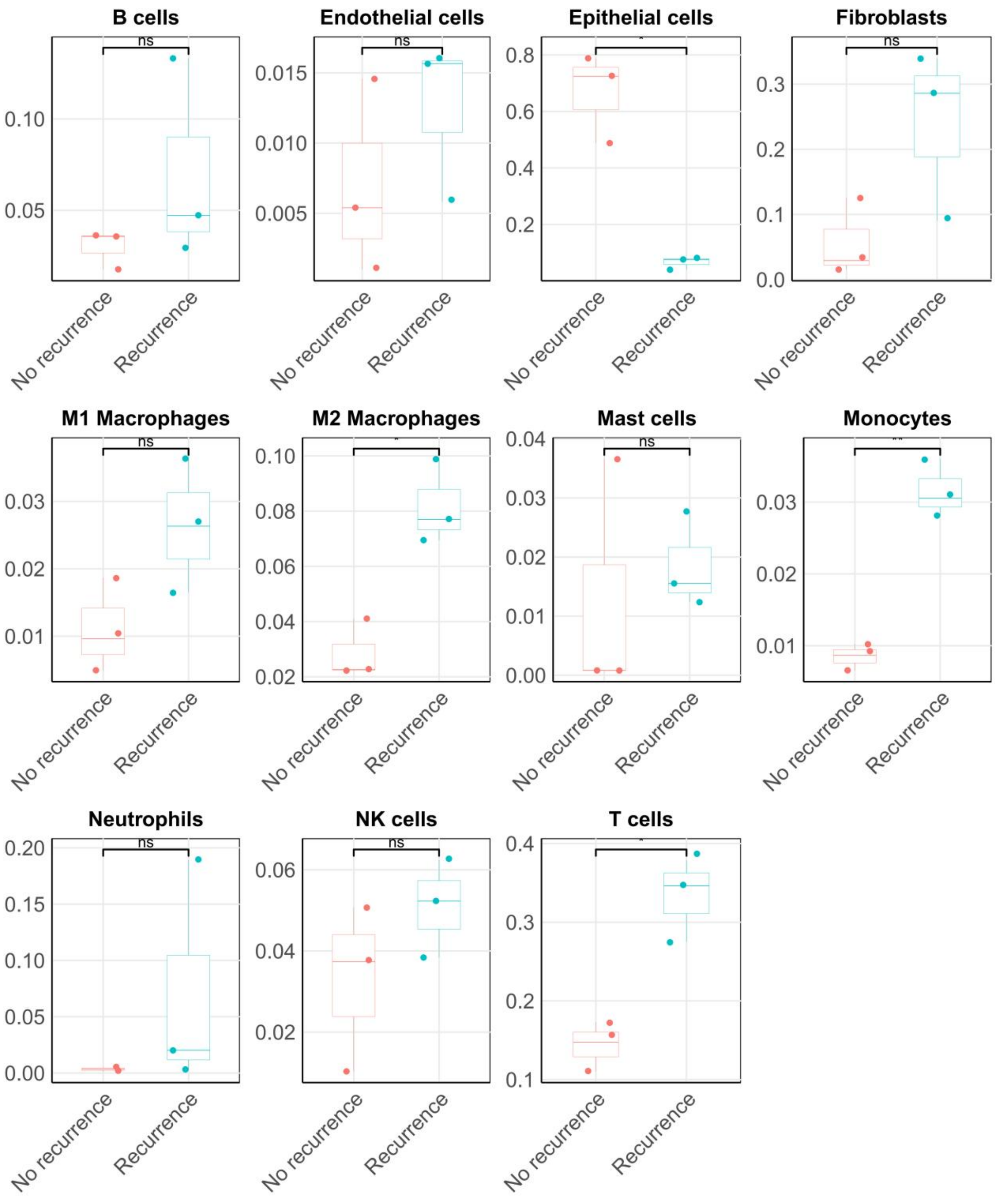

Supplementary figure 6. The association between immune cells and tumor recurrence in cervical cancer revealed by single-cell analysis.

Supplementary table 1. Kaplan-Meier survival analysis of key prognostic genes in our cohort.

Gene name	p value (OS)	p value (PFS)	Association with survival
GGT6	0.062	0.0076	Low expression: poorer PFS
PRR4	Not significant	0.033	High expression: poorer PFS
MYT1L	0.062	0.062	Not significant
ALOX12	Not significant	0.085	Not significant
C1orfB7	0.042	Not significant	High expression: poorer OS
FRG2	0.028	Not significant	Low expression: poorer OS
DPYBL5	0.062	Not significant	Not significant
H1_7	0.062	Not significant	Not significant

Supplementary table 2. Chemotherapy-related adverse events.

Adverse Effect	Number
**Hematologic**	51 (98.1)
G3-4	15 (28.8)
Neutropenia	43 (82.7)
G3-4	13 (25.0)
Thrombocytopenia	27 (51.9)
G3-4	7 (13.5)

Anemia	45 (86.5)
G3-4	3 (5.8)
**Nonhematologic**	
Nausea/vomiting	42 (80.8)
Hepatotoxicity	15 (28.8)
Infusion reaction	1 (1.9)
Hypothyroidism	0 (0)

Supplementary table 2. Cadonilimab-related adverse events.

Adverse Effect	Number (%)
**Hematologic**	0 (0)
**Nonhematologic**	
Hepatotoxicity	4 (7.7)
Infusion reaction	4 (7.7)
Rash	8 (15.4)
Hypothyroidism	26 (50.0)
